# Tools for the investigation of adverse events: scoping review^*^

**DOI:** 10.1590/1980-220X-REEUSP-2021-0519en

**Published:** 2022-06-10

**Authors:** Lucas Rodrigo Garcia de Mello, Barbara Pompeu Christovam, Ana Paula Amorim Moreira, Erica Brandão de Moraes, Graciele Oroski Paes, Cassiana Gil Prates

**Affiliations:** 1 Universidade Federal Fluminense, Escola de Enfermagem Aurora Afonso Costa, Niterói, Rio de Janeiro, Brazil.; 2 Universidade Federal Rio de Janeiro, Escola de Enfermagem Anna Nery, Rio de Janeiro, RJ, Brazil.; 3 Hospital Ernesto Dornelles, Serviço de Epidemiologia e Gerenciamento de Riscos, Porto Alegre, RS, Brazil.; 4 Centro Brasileiro para o Cuidado à Saúde Informado por Evidências: Centro de Excelência do Instituto Joanna Briggs, São Paulo, SP, Brazil.

**Keywords:** Patient Safety, Risk Management, Patient Harm, Health Quality Management, Safety Management, Seguridad del Paciente, Gestión de Riesgos, Daño del Paciente, Gestión de la Calidad en Salud, Administración de la Seguridad, Segurança do Paciente, Gestão de Riscos, Dano ao Paciente, Gestão da Qualidade em Saúde, Gestão da Segurança

## Abstract

**Objective:**

To map, in the literature, the risk management tools aimed at investigating health adverse events.

**Method:**

Scoping review according to the *Joanna Brigss Institute*, with acronym PCC (Population: hospitalized patients, Concept: tools for the investigation of adverse events, and Context: health institutions) carried out in MEDLINE (OVID), EMBASE, LILACS, Scopus, CINAHL, and gray literature.

**Results:**

The search totaled 825 scientific productions, 31 of which met the objective of the study, which consisted of 27 scientific articles and 4 expert consensus. It was possible to carry out a synthesis of the necessary steps for the investigation of adverse events and use of the tools according to the extent of damage.

**Conclusion:**

The practice of investigating adverse events should be guided by a thorough understanding of contributing factors, a fair culture, and the involvement of senior leadership.

## RESUMO

**Objetivo:**

Mapear na literatura as ferramentas da gestão de risco voltadas para investigação de eventos adversos na saúde.

**Método:**

Revisão de escopo segundo o *Joanna Brigss Institute*, com acrônimo PCC (População: pacientes internados, Conceito: ferramentas para a investigação de eventos adversos e Contexto: instituições de saúde), realizada nas bases MEDLINE (OVID), EMBASE, LILACS, Scopus, CINAHL e literatura cinzenta.

**Resultados:**

A busca totalizou 825 produções científicas, sendo que 31 atenderam o objetivo do estudo, sendo composta por 27 artigos científicos e 4 consensos de especialistas. Foi possível realizar uma síntese das etapas necessárias para a investigação de eventos adversos e utilização das ferramentas de acordo com o grau do dano.

**Conclusão:**

A prática de investigação de eventos adversos deverá ser pautada na compreensão exaustiva dos fatores contribuintes, cultura justa e envolvimento da alta liderança.

## RESUMEN

**Objetivo:**

Mapeo en la literatura de las herramientas de la gestión de riesgo con énfasis en la investigación de eventos adversos en salud.

**Método:**

Revisión de alcance según *Joanna Brigss Institute* con el acrónimo PCC (Población: pacientes ingresados, Concepto: herramientas para la investigación de eventos adversos y Contexto: instituciones de salud) realizada en las bases de datos MEDLINE (OVID), EMBASE, LILACS, Scopus, CINAHL y literatura gris.

**Resultados:**

La búsqueda llegó a un total de 825 producciones científicas, siendo que 31 lograron el objetivo del estudio, el cual fue compuesto por 27 artículos científicos y 4 consensos de expertos. Fue posible realizar una síntesis de las etapas necesarias para la investigación de eventos adversos y utilización de las herramientas de acuerdo con el grado del daño.

**Conclusión:**

La práctica de investigación de eventos adversos deberá pautarse en la comprensión exhaustiva de los factores contribuyentes, cultura justa e involucramiento de alto liderazgo.

## INTRODUCTION

In 2013, from the publication of the Resolution of the Collegiate Board of Directors – RDC no. 36/2013, it was possible to understand that risk management is a form of proactive and reactive approach to the risks that the patient runs in the health services^(1,2)^.

The construction of the concept and the practical applicability of risk management has its origins in the industry and aviation segments. Moreover, activities related to this topic represent a proactive approach to identified risks, insofar as they allow the identification, planning, and implementation of actions and activities that work as barriers to prevent a risk from resulting in an incident^(3)^.

In Brazil, in 2013, the Ministry of Health (MS) launched the National Patient Safety Program (*PNSP*), through the publication of Ordinance No. 529, of April 1. PNSP aims to prevent, monitor, and reduce the incidence of adverse events (AE) in the care provided, promoting continuous improvement related to patient safety^(2)^.

A study carried out in Brazil showed an incidence of 7.6% adverse events, of which 66.7% were preventable. Thus, the incidence of patients with adverse events in the three hospitals included in the study was similar to that of international studies; however, the proportion of preventable adverse events was considerably higher in Brazilian hospitals^(4)^.

The investigation of adverse events in health services, considered a requirement of the *PNSP*, is a fundamental action to identify and map the failures occurring in assistance and explore the possible causes leading to the incident, and devise action plans to allow the reduction of the level of damage and the prevention of a possible recurrence^(1–4)^.

Therefore, health institutions shall be aware of the challenges imposed by patient safety, such as that of developing a more careful investigation regarding the error and harm patients experience. Because immediately after an incident, people make quick judgments and very often blame the person most obviously connected with the disaster^(2,3)^.

Currently, there are tools and/or instruments to help in the investigation, conducting a robust analysis and reaching consistent results. The most used tools for investigation of AE in health are: Root cause analysis with contributing factors adapted *from Three levels of RCA investigation*; *Human Factors Analysis and Classification System* (HFACS); *Canadian Incident Analysis Framework*; *Yorkshire Contributory Factors Framework* and the London Protocol. However, in the midst of this variety of instruments, many institutions make the mistake of selecting a complex tool, or perhaps one not suitable for the investigation process, where the manager him/herself has difficulty conducting the operationalization^(3,5,6)^.

Therefore, it is necessary to explore tools aimed at investigating adverse health events. Furthermore, since the implementation of the reactive risk management methodology in healthcare organizations, there has been a reduced number of tools that fully serve the healthcare sector and which take all the steps required to complete the root cause analysis and the identification of all contributing factors to the elaboration of an efficient improvement plan.

This study aims to map, in the literature, the risk management tools focused on the investigation of health adverse events.

## METHOD

### Design of Study

This is a scoping review aimed at mapping the literature in a particular field of interest, identifying and exploring the nature of the productions and allowing the synthesis of existing scientific evidence related to the theme, in addition to identifying gaps in research knowledge, especially when reviews on the topic have not yet been published. The review was developed based on the recommendations of the *Joanna Briggs Institute* (JBI)^(5)^. The research question was based on the acronym PCC (Population, Concept and Context): what tools are used in patient safety to investigate health adverse events? The term Population refers to inpatients; Concept, to tools for the investigation of health adverse events, and Context, to health institutions.

### Eligibility Criteria

From the PCC acronym, this review population were patients hospitalized due to any pathologies. Thus, studies involving hospitalized patients in any inpatient unit in a health institution were included. Regarding the concept, studies addressing the tools for investigating health adverse events were included. They are techniques or instruments that aim to identify and analyze the root cause of healthcare-associated unnecessary harm. Studies describing one or other tools to investigate adverse events based on root cause analysis were included. Finally, in the context, studies with patients hospitalized in a health institution were included.

Therefore, the types of sources this review considered were descriptive and analytical observational studies, individual case reports, expert consensus, guidelines, protocols, secondary studies, dissertations, and theses. Language filters and time periods were not applied. However, editorials, abstracts, correspondence, monographs, reviews, articles that were not available in full in the data sources were excluded. The searches were carried out in November 2020.

### Search Strategy

**Chart 1.**
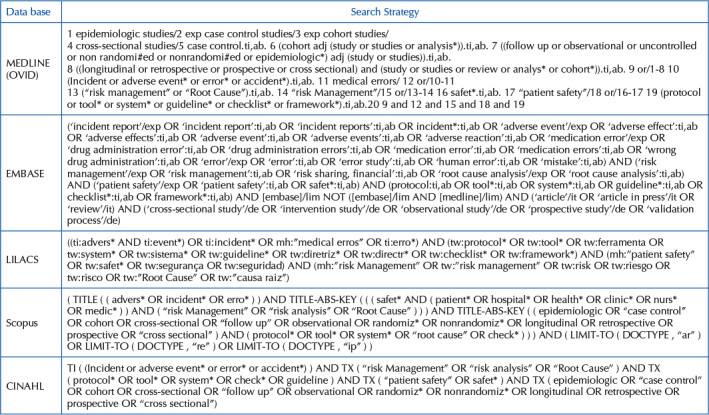
Databases and respective search strategies – Niterói, RJ, Brazil, 2020.

According to JBI guidelines, the search strategy took place in three stages. In the first one, a limited search on the subject was carried out on the PubMed electronic database, on the Mesh and CINAHL platforms, to identify the descriptors most commonly used in the literature. In the second stage, the research was carried out in the following information bases: MEDLINE (OVID), EMBASE, LILACS, Scopus, and CINAHL, as shown in Chart 1.

In the third stage, the gray literature was consulted using the repository of the Brazilian Digital Library of Theses and Dissertations (*BDTD*), made available by the Ministry of Science, Technology and Innovation. In addition, searches were carried out in the agencies and foundations for Patient Safety to identify manuals and expert consensus on the investigation of adverse events.

### Source Selection

The records were imported into a reference manager for information management (EndNote Web). Duplicate studies were considered only once. The study selection process was performed by two independent reviewers, and discrepancies were resolved by a third reviewer.

**Figure 1.**
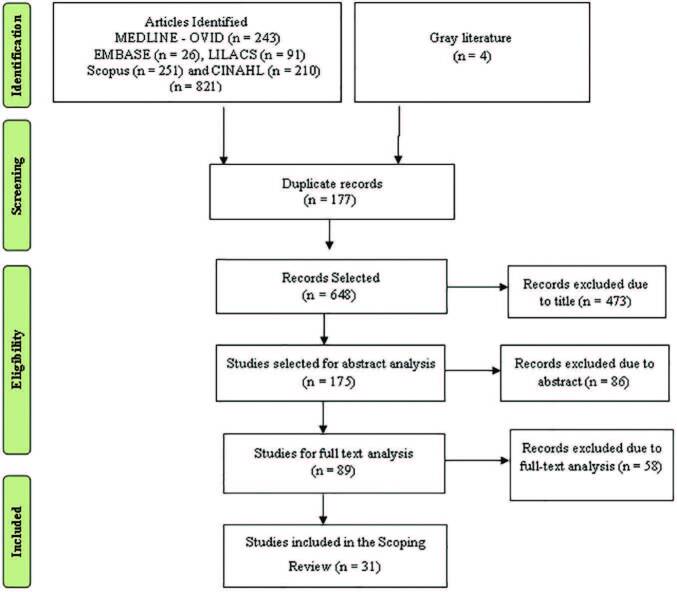
Flowchart *Preferred Reporting Items for Systematic Reviews and Meta – Analyzes Extension for Scoping Reviews* (PRISMA-SCR) on the selection of studies, Niterói, RJ, Brazil, 2020.

The selection was carried out in two stages. The first stage consisted of reading and evaluating the titles and abstracts of the records found through the search strategy, with potentially eligible studies having been pre-selected. In the second stage, the full text of the pre-selected studies was evaluated to confirm their eligibility (Figure 1). Subsequently, the two reviewers independently and blindly read the titles and abstracts to reduce the possibility of interpretative bias. Then, in the event of disagreement at this stage, a third reviewer was consulted to analyze the record and guarantee the resolution through a consensus meeting for inclusion or exclusion in the study.

### Data Extraction and Items

For the process of extracting eligible articles, the instrument developed by the JBI was used as a basis, which contained the following topics: year of publication, authorship, journal/ institution, title, study objective, methodology, country of study, and type of publication. In each publication, the tools used to investigate adverse events, the strengths in the application found by the authors, the problems and limitations described, and the recommendations for use were identified and extracted^(5)^. Study selection steps were carried out according to the scoping review flowchart (PRISMA – ScR).

### Presentation of Results

The extracted data were presented in the form of tables and figure, to align with the objective of this scoping review. The tables included data about the year of the study, authorship, title, design of study, and a description of the techniques, tools, and instruments used to investigate AE. A figure was created describing a synthesis of the findings of the review, allowing the creation of an important and necessary “guide” for the selection of tools and/or techniques to conduct the investigation process according to the extent of damage initially detected. This way, describing how the results were related to the objective and question of the review.

### Ethical Aspects

As it is an investigation whose method consists of a scoping review, the present study was not submitted to the Research Ethics Committee of the Universidade Federal Fluminense. However, Resolution No. 466/12, of the National Health Council, was followed with regard to the analysis and sharing of study results.

## RESULTS

The searches resulted in 825 scientific productions distributed in the databases. Figure 1 presents the stages of the study and the results obtained, consisting of 27 articles and four manuals and expert consensus, totaling 31 studies.

**Chart 2.**
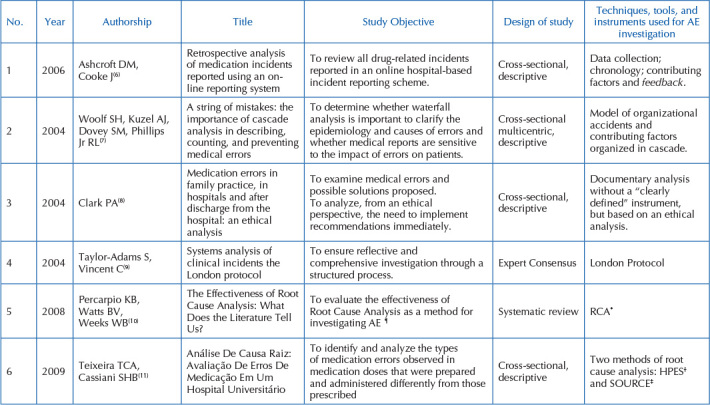

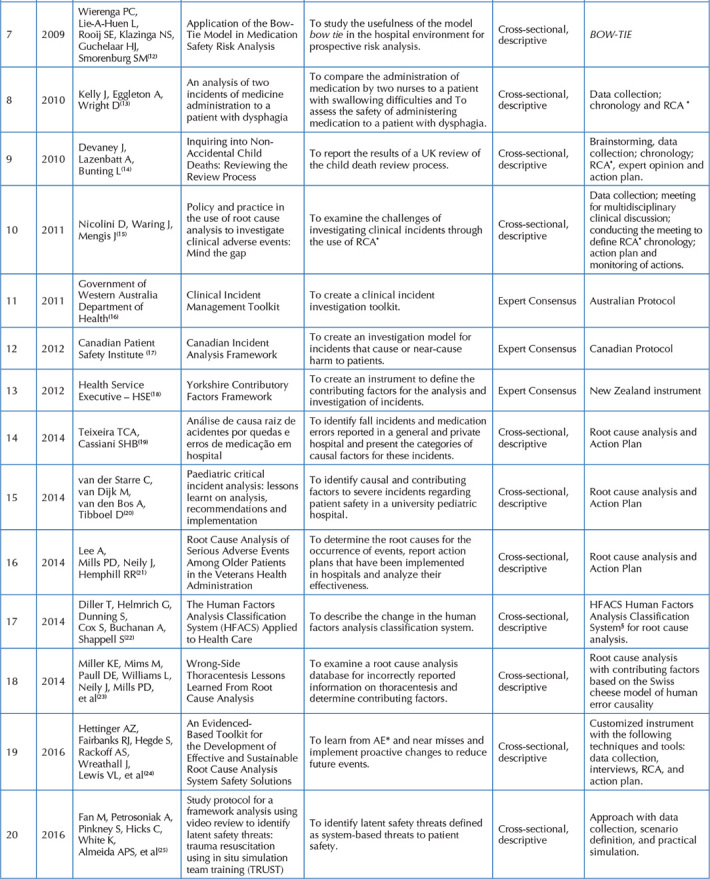

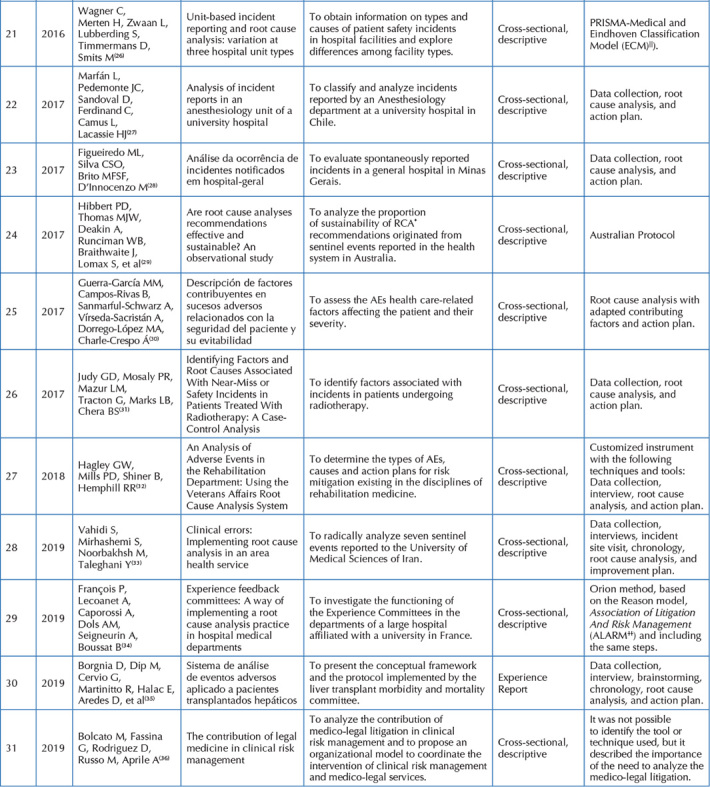
Description of studies included in the review – Niterói, RJ, Brazil, 2020.

Chart 2 shows the authors, year of publication, design of study, study objectives, as well as the instrument used or described by the authors^(6–36)^. When analyzing the origin of the studies, it was evident that they were carried out in different continents, being predominant in Europe, with 11^(6,9,12,13,14,15,20,26,30,34,36)^ studies (35.48%) and North America, with 12^(7,8,10,17,21–25,31,32,35)^ studies (38.70%), South America totaling four^(11,19,27,28)^ studies (12.90%), and finally the Asian continent with four^(16,18,29,33)^ studies (12.90%).

*RCA = Root Cause Analysis; ^†^HPES = *Human Performance Enhancement System*; ^‡^SOURCE = *Seeking Out the Underlying Root Causes of Events*; ^§^HFACS = *Human Factors Analysis and Classification System*; ^||^ECM *= Eindhoven Classification Model*; ^¶^AE = Adverse Events; **WHO = World Health Organization; ^††^ALARM = *Association of Litigation and Risk Management.*

In addition, it was possible to highlight the interest and growth of research on the subject, with emphasis on the years 2014–2019. It is important to point out that in 2004, in Europe, the tool entitled London Protocol was published^(9)^ and then only in 2019, also in Europe, was the first study released^(34)^ using the *Association of Litigation And Risk Management* based on *Reason model*. As for the method used, twenty were qualitative, four were quantitative studies, four were expert consensus, one was a systematic review, one was an experience report, and one was a study with mixed methods.

**Figure 2.**
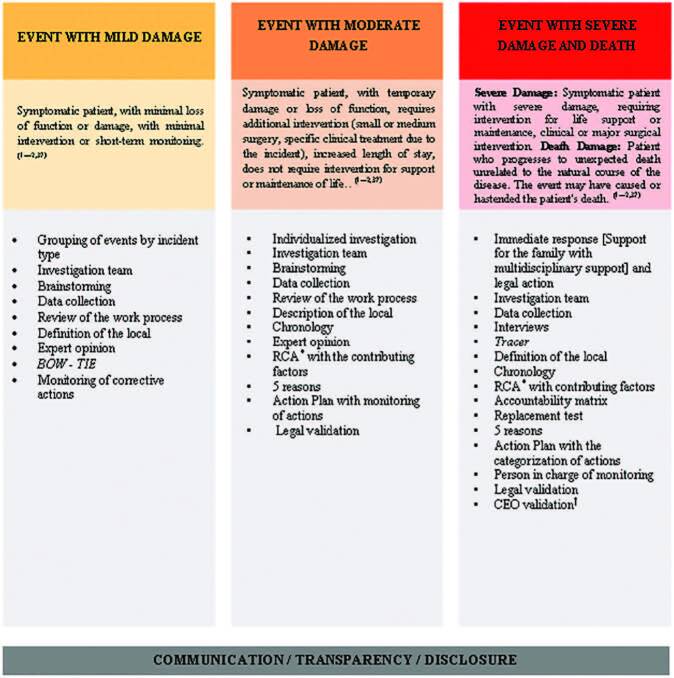
Synthesis of techniques and tools used in the investigation according to the extent of damage, Niterói, RJ, Brazil, 2020.

In Figure 2, it was possible to establish a synthesis of the review findings, allowing the creation of an important and necessary “guide” for the selection of tools and/or techniques to conduct the investigation process according to the degree of damage initially detected. In addition, the “guider” demonstrates the need for effective communication among the different levels of the organization, transparency in monitoring the investigation, and finally resulting in the practice of *disclosure*.

*RCA = Root Cause Analysis; ^†^CEO = Chief Executive Officer.

## DISCUSSION

This review gathered information about the tools for investigating health adverse events, especially what instruments and techniques were applied and the results obtained. From this review, it was possible to identify the tools used to investigate AEs, such as *Bow tie*, ACR with contributing factors, 5 reasons, accountability matrix, and action plan; in addition, the techniques and instruments such as interviews, data collection, chronology and the methodology *tracer* itself.

It is important to highlight the definitions of each of the tools identified in this review. *Bow Tie* was originally created for risk identification; however, it allows the investigation of the possible causes that led to the AE and still establish contingency actions^(1–7)^. On the other hand, RCA with contributing factors allows the reconstruction of the logical sequence of factors that favored the occurrence of the incident in a systematic way. The 5 reasons tool allows the identification and investigation of the possible causes that led to the incident, based on the problem, using the five questions^(8–12)^.

In the literature, it is observed that all studies used a tool to identify and categorize the contributing factors aiming at root cause analysis, since this step allows the investigator to identify all the factors that contributed to the occurrence of AE^(8,10,13–22)^.

In several studies, the authors referred to the effectiveness of RCA, using quantitative and qualitative measures, as well as knowledge based on clinical experience. However, it reinforces the need to exhaustively apply this method, besides creating a database of contributing factors^(23–30)^.

In some authors’ opinion, the performance of an RCA varies from institution to institution, due to the lack of standardization and minimal attention to reliability among evaluators and intra-evaluators, thus leading to findings driven by personal behaviors and the inconsistent identification of systematic errors^(10,21,29,31,32,33,34,35,36,37,38,39,39,40)^.

Furthermore, an RCA that only focuses on “what happened?” and “who was responsible?”, rather than identifying the real root causes that define the “why?” the event occurred, allows a culture of guilt in which the health professional is formally or informally punished, instead of identifying the impact on the patient, the employee, and the institution. Even the Canadian investigation model begins with the “Preparation for Analysis” stage, thus consisting of a preliminary investigation aimed at determining the appropriate follow-up of an incident, including the need for analysis; an initial investigation or fact-finding is required. The main outcome of this step will be the construction of a high-level chronology and documentation of known facts related to the incident^(17)^.

Another point that draws attention in the studies is the interview stage. The use of interviews is a limited method, but it is the most used tool compared to observation or *tracer* ^(30)^. This practice cannot be the only one used, as it weakens the RCA strength, as employees can present biased speeches and report what “should have happened” and not what actually happened. However, observation techniques, auditing of the therapeutic itinerary, *in loco*, collaborate with the investigation stage and the exclusion of professionals’ individual attitudes^(25,41)^.

Therefore, the *tracer* is the method most used as an evaluation mechanism in the accreditation processes in health institutions, thus allowing the identification of conformities and non- conformities and even incidents, in line with established standards and requirements, resulting in the evaluation of the quality of care practices and aspects related to patient safety^(25,41)^.

Another point, strongly recommended, is the use of the accountability matrix, with the objective of guiding actions based on the detection related to the professional’s factor as a contributor to the occurrence of the incident or influence on the extent of damage^(25,39,42)^.

According to the *Agency for Healthcare Research & quality* (AHRQ), from a just culture, frontline professionals are comfortable reporting incidents related to patient safety, including their own, while maintaining their professional responsibility. Thus, in the constant search for excellence and patient safety, health institutions implemented the matrix proposed by the *National Patient Safety Agency* (NPSA)^(25,39,43)^.

According to several studies on this topic, an error, based on the professionals’ factors, specifically on their professional ability, occurs when they are involved in a task that is very familiar to them or commonly practiced in their work routine. In the hospital setting, professionals often perform repetitive tasks that require attention; however, these seemingly automatic practices and behaviors are particularly susceptible to attention or memory failures, especially if someone is interrupted or distracted during the process^(21,23,34,38,39)^.

However, sometimes, errors can also occur when professionals consciously do not perform or do not follow the previously defined flow, as they do not consider it as a risk prevention barrier that could result in damage, thus resulting in a violation. This phenomenon is the result of intentional deviations from accepted practices. The failure mode in this case is intentional, that is, the individual knew the accepted practice and still chose to ignore it^(18,31,38,42)^.

In addition, routine violations in many segments tend to be habitual in nature and are generally permitted by institutions that tolerate *rule bending.* This way, they become ingrained in the professionals’ culture and habits. In the hospital setting, this is often manifested by routine failure to follow policy or by the development of an alternative solution to a process or task; in fact, many professionals do not identify this as an intentional act^(12,24,28,31,43,44,45)^.

In this context, it is important to highlight that the London protocol applies the Organizational Accident model proposed by James Reason, in which he emphasizes that the analysis shall have a much broader understanding of the cause of the incident, with less focus on the professional and/or individual who made a mistake, and more on systemic organizational factors existing in the institution^(9)^.

Several studies point out that institutions with a positive culture are characterized by communications based on mutual trust, a shared perception of the importance of safety and trust in the effectiveness of prevention measures; above all, they recognize the differences between human error, negligence, violation, and reckless conduct^(10,30,39,40)^.

However, the operationalization of the method cannot be based only on the steps of data collection, interviews and chronology, because as mentioned above, these steps may still undergo human interference. Therefore, the recommendation is to use the observation technique, more specifically a *tracer*, plus practical simulation of the processes, techniques and/or routines being examined^(25,30,41)^.

Other studies have emphasized the need for validation of the *Chief Executive Officer* (CEO), as the highest authority of the organization, with the objective of stimulating communication and the certainty that this topic will be seen with the same degree of importance as, for example, financial results, but also ensuring that these actions were carried out^(10,39,43)^.

Finally, the need for the institution’s legal department to actively participate in this process. According to one of the studies, the analysis of medico-legal disputes proves to be an excellent tool with high precision and reliability for the detection of situations previously not recognized and/or not recorded in the investigation process by the responsible team.^(31)^.

In none of the analyzed studies, it was evidenced that the analysis and investigation of events come from a single model. The operationalization of this practice is guided by numerous tools and instruments built for this purpose. For instance, the root cause analysis and action plan were adapted to the reality of the health segment and/or for institutional applicability^(8,9,12,15,20,26,29,35,36,45,46,47,48,49)^.

## STUDY LIMITATIONS

As limitations, despite efforts to develop a comprehensive search strategy, some aspects related to methodological procedures stand out, such as the number of selected databases, non-availability of the study full text. In addition, despite advances in health research on the tools used to investigate AEs, there are still limitations arising from the lack of studies with a high level of evidence, such as randomized clinical trials, systematic reviews with meta-analysis to assess the effectiveness of the tools for the investigation of AEs in health, and concentration of the most used tools in clinical practice, classified as gray literature. However, in spite of the existing scientific gap, arising from the fact that quality tools come from other segments other than health, this study is justified.

### Contributions to Health-Related Research

Due to the need of in-depth analysis of this object of study, which is fundamental for the continuous improvement of health organizations, aiming to help filling the gap in the literature on this subject, this study is a great contribution. It is based on the provision of an analysis of studies on the tools used to investigate AEs, contributing to the improvement of work processes, especially in patient safety centers in the practice of investigating adverse events, resulting in an increase in the quality of care provided to the population.

## CONCLUSION

The study identified scientific publications on tools and techniques for investigating adverse health events, highlighting the importance of a model based on a thorough understanding of the contributing factors to the occurrence of AE. The main measure is the use of a robust RCA method that allows identification and categorization of these factors.

It was evident that the interview, an extremely used technique, shall be complemented with other methods, such as the method *tracer*, to ensure the understanding of latent and active failures in clinical practice operated by the workers, allowing a systemic view of the work process.

The need to apply the accountability matrix should be noted, as it allows the increase of the AE management process, based on a fair culture, feeding the system back to a model based on the sharing of responsibilities at all levels of the organization.

The importance of involvement and active participation of senior leadership, especially the CEO of the organization, shall be highlighted, with the objective of equating the Patient Safety issue at the same level as the institution’s financial results, considering that the organization’s sustainability is directly related to quality of care, patient experience, value-based health.

## ASSOCIATE EDITOR

Cristina Lavareda Baixinho

## Apoio financeiro

 Conselho Nacional de Desenvolvimento Científico e Tecnológico (CNPq). Processo: 133103/2019-6.
